# The Effects of Weather and Fertilization on Grain Yield and Stability of Winter Wheat Growing on Orthic Luvisol—Analysis of Long-Term Field Experiment

**DOI:** 10.3390/plants11141825

**Published:** 2022-07-12

**Authors:** Lukáš Hlisnikovský, Peter Ivičic, Przemysław Barłóg, Witold Grzebisz, Ladislav Menšík, Eva Kunzová

**Affiliations:** 1 Department of Nutrition Management, Crop Research Institute, Drnovská 507, Ruzyně, 161 01 Prague 6, Czech Republic; ivicic@vurv.cz (P.I.); ladislav.mensik@vurv.cz (L.M.); kunzova@vurv.cz (E.K.); 2 Department of Agricultural Chemistry and Environmental Biogeochemistry, Poznan University of Life Sciences, Wojska Polskiego 71F, 60-625 Poznan, Poland; przemyslaw.barlog@up.poznan.pl (P.B.); witold.grzebisz@up.poznan.pl (W.G.)

**Keywords:** *Triticum aestivum* L., temperature, precipitation, mineral fertilizers, farmyard manure, non-linear response models, climate change

## Abstract

Based on a long-term experiment in Prague, established in 1954, we analyzed the effect of weather and seven fertilization treatments (mineral and manure treatments) on winter wheat grain yield (GY) and stability. In total, 23 seasons were analyzed, where a wheat crop followed a summer crop of potatoes. A regression analysis showed that, since the experiment started, there has been a significant increase in the annual daily maximum, average, and minimum temperature of 0.5 °C, and an increase in annual rainfall of 0.3 mm. Grain yield was positively associated with April precipitation, mean daily temperature in October, and daily maximum temperature in February. Yields were most stable between years with two fertilizer treatments that supplied a mean of 47 kg N ha^−1^yr^−1^, 54 kg P ha^−1^yr^−1^, and 108 kg K ha^−1^yr^−1^. The rate of N at which grain yield was optimized was determined according to the linear-plateau (LP) and quadratic response models as 44 kg N ha^−1^yr^−1^ for the long-strawed varieties and 87 kg N ha^−1^yr^−1^for short-strawed varieties.A gradual increase in yields was observed in all treatments, including the unfertilized control, which was attributed to improved varieties rather than to a changing climate.

## 1. Introduction

Wheat is the most widely grown cereal in the EU, withan estimated production of 130 million tonnes in 2021. Wheat production, grain yield (GY), and its quality areinfluenced by a wide range of variables, mainly by the soil–climate conditions and fertilization. Other factors, such as the preceding crop in the crop rotation [1,2], wheat variety [3], or tillage practices [4], also play a significant role in yield formation and stability.

The effect of weather on agricultural productivity has been analyzed for a long time [5]. In the context of weather, climate change is currently the most highlighted term. Its precise definition can be problematic [6], but generally, it refers to long-term climate shifts in temperature and precipitation patterns, directly affecting short-term interannual weather variation (more frequent occurrence of extraordinary weather circumstances during the growing seasons). Climate change is a long-term and gradual process. There is a wide range of papers analyzing the impacts of ongoing climate change on future agricultural production based on different models. These papers, analyzing the relationship between changing climatic conditions and crop production, have been published since the early 1990s [7,8,9] and continue to this day, confirming that climate change is significantly affecting and will continue to affect crop yields and quality, positively or negatively (according to the locality), and that different regions will be affected to a different extent [10,11,12]. In England, for example, conditions for growing wheat can be expected to remain favorable until the middle of the 21st century, without the long periods of drought that are, on the other hand, expected in some European countries [13]. In Spain, conditions for growing wheat can be expected to remain favorable, while conditions for sunflower are likely to deteriorate in the future due to climate change [14]. In the Czech Republic, climate change is connected with more frequent occurrence of intensive dry episodes [15]. For example, a severe drought was recorded in the 2011–2012 season in south Moravia region, resulting in the lowest cereal yields in the past 52 years [16,17]. Severe wheat yield losses were also recorded in France, Belgium, and Switzerland in 2016, when mean wheat yields decreased significantly in comparison with the previous ten years due to either drought or abnormally wet conditions [18]. The exact impact of climate change on crop production is difficult to assess. Long-term field trials can provide some insight, as they are well defined, include different and controlled fertilizer treatments, and detailed weather observations are made at the trial site. Fertilization represents one of the tools that help balance the effects of unpredictable and rapidly changing weather conditions.Thanks to data from long-term experiments, we can analyze the effect of weather in each month on crop yields, the effect of different fertilizer regimes on yield and its stability, and the recommend optimal fertilizer rates for selected soil–climate conditions. A study of a long-term experiment at Müncheberg (northeast Germany) showed that the effect of weather on spring barley yields was major, accounting for 55% of variation, so yields are strongly dependent on the weather conditions of the year. The Bayesian linear regression analysis showed that yield was influenced mostly by rainfall between April and July, especially for treatments fertilized with high rates of mineral N. Fertilizer application had a much smaller effect on yield, accounting for 11% of variation. Mineral N application also reduced year-to-year variation in yield [19]. A similar study of long-term effects of weather and fertilization on the yields of winter wheat at Rothamsted showed a correlation between winter wheat yield and mean temperature in November, April, and May, and precipitation in October, February, and June. Yields of spring barley were correlated with mean temperature between February and June, and with precipitation from April to July and in September [20]. An analysis of two long-term experiments in Germany showed that barley grown in crop rotations dominated by cereals proved a yield with lower stability and higher production risk than rotations with higher crop diversity. The highest yield stability of barley was reached with a medium N rate (70 kg N ha^−1^yr^−1^) [21,22].

Inspired by the above-mentioned papers, we decided to analyze the long-term field experiment (LTE) in Prague, established in 1954. The aim of our work is to analyze whether the yield of winter wheat is controlled by weather conditions, which month conditions (max. temperature, mean temperature, min. temperature, precipitation) significantly influence the yield, what type of fertilization provides stable yields, and what fertilizer dose represents the optimum under given soil and climatic conditions.

## 2. Results

### 2.1. Yield Development

**Figure 1:**
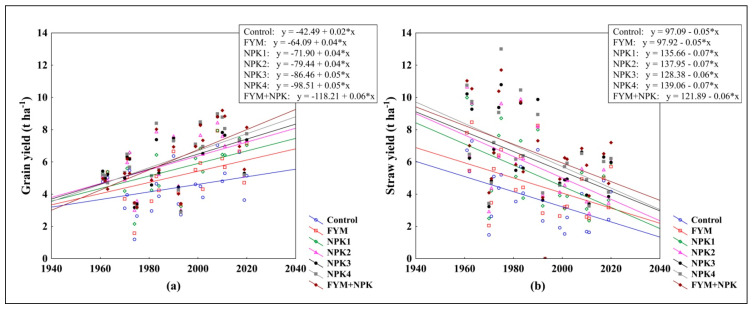
The development of (**a**) grain yield, GY (t ha^−1^) and (**b**) straw yield, STY (t ha^−1^) from 1961 to 2020, as affected by the fertilizer treatment.

Winter wheat GY increased since the start of the LTE for all fertilizer treatments, including the unfertilized control. The average seasonal GY increase for control, FYM, NPK1, NPK2, NPK3, NPK4, and FYM+NPK was approximately 24, 35, 40, 43, 47, 53, and 63 kg ha^−1^, respectively (Figure 1a). As the short-strawed wheat varieties began to be grown in 1983, the straw yield (STY) has been declining (Figure 1b).

### 2.2. Weather Conditions

**Figure 2:**
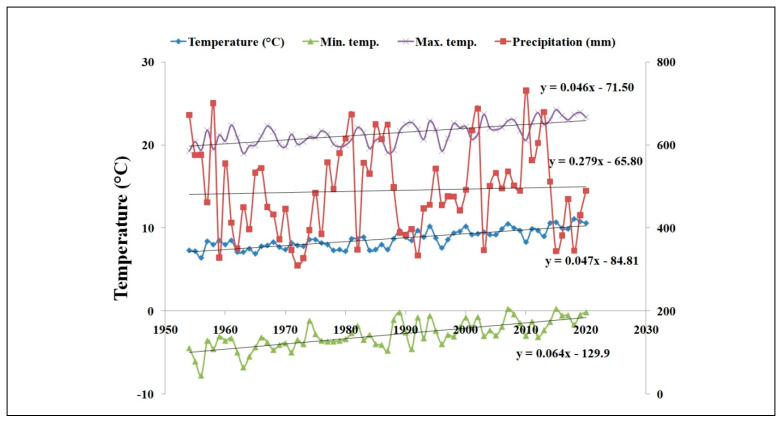
The development of average, min., max. temperatures and precipitation, LTE Prague, 1961–2020.

The average daily maximum temperature averaged over the years increased by 0.05 °C from 1954 to 2020, while the average daily temperature increased by 0.05 °C, minimum temperature by 0.06 °C, and precipitation increased by 0.3 mm yr^−1^ (Figure 2). In summary, the site of the LTE has been gradually warming since 1954, and the amount of rainfall increased slightly.

**Figure 3:**
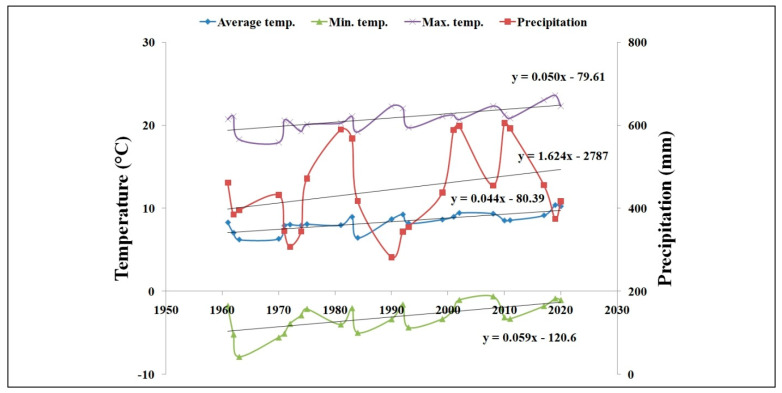
The development of average, min., max. temperatures and precipitation, LTE Prague, 1961–2020 (data from 23 seasons that are analyzed in this paper).

An equivalent analysis of growing seasons for which there were corresponding wheat yield data analyzed in this paper (1961 to 2020) also showed similar trends. The daily maximum, average, and minimum temperatures increased by 0.05 °C, 0.04 °C, and 0.06 °C, respectively, while precipitation increased by 1.6 mm yr^−1^ (Figure 3).

### 2.3. Relationship between Weather and Yields

**Figure 4:**
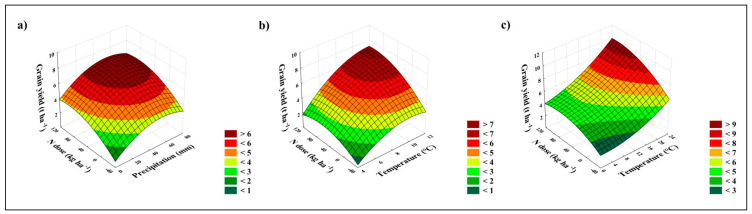
Response surface of the effect of applied N (kg ha^−1^ N) on winter wheat GY (t ha^−1^) as affected by (**a**) April rainfall, (**b**) average October temperature, (**c**) max. February temperature.

To answer the question of whether the weather differed significantly between periods with different wheat varieties, we used ANCOVA, where GY, STY, temperature, and precipitation served as covariates. According to the results, the temperature did not differ significantly between the periods when each variety was grown (*d.f.* = *8, F* = 2.2, *p* = 0.114). In the case of precipitation, we observed a significant difference (*d.f.* = 8, *F* = 2.9, *p* < 0.05) between the period when the Zdar variety was grown (lowest mean precipitation, 327 mm, mean value over three seasons, 1990, 1992, and 1993) and the period when the Alka variety was grown (highest mean precipitation, 593 mm, mean value over two seasons, 2001 and 2002). These two periods were the only ones significantly different (in terms of rainfall). There were no significant differences in rainfall between the other periods. From this point of view, the general effect of increasing temperatures and precipitation on gradually increasing yields is so far insignificant, and the increasing trend in GY is mainly due to the use of modern wheat varieties. The conditions of a particular year affect yields significantly. We used multiple linear regression (MLR) and correlation to investigate the relationship between the weather and yield. The MLR answers the question of whether yields can be predicted from the temperature or rainfall in a particular month of the growing season. We evaluated the relationship between the rainfall, max., average, and min. temperatures and between the average GY (all fertilizer treatments together) and the GY of each fertilizer treatment (separately—control, FYM, NPK1...FYM+NPK). The results of the MLR were not statistically significant in any case (n = 32), as the p value ranged from 0.07 to 0.9. On the other hand, the results of the correlation analysis were already significant. In the case of rainfall, we found a positive significant moderate correlation between GY and rainfall in April for all fertilizer treatments (r = 0.5). According to Figure 4a, the highest wheat yields can be expected when 50 to 60 mm of rain falls in April. There was also a significant positive correlation between GY and average temperature in October (r = 0.5, positive moderate correlation, Figure 4b) and maximal temperature in February (r = 0.6, positive moderate correlation, Figure 4c). Higher yields were therefore associated with wetter conditions in April up to a maximum of 60 mm month^−1^ and warmer conditions in October and February. However, the quadratic function in Figure 4b is approaching its maximum, and a further increase in temperature could lead to a decrease in yield in the future.

### 2.4. Yield Stability

According to Kang’s ranksum statistics, in which Shukla’s stability variance was used to identify high-yielding and stable fertilizer treatments, the highest stability of long-strawed wheat varieties was provided by the NPK3 treatment (rank 1), followed by NPK2 (rank 2), NPK4 (rank 3), NPK1 (rank 4), FYM+NPK (rank 5), FYM (rank 6), and control (rank 7) treatments. For the short-strawed wheat varieties, the most stable yields were provided by the NPK2 and NPK3 treatments (rank 1), followed by NPK 4 (rank 3), FYM+NPK (rank 4), NPK1 (rank 5), FYM (rank 6), and control (rank 7) treatments. Treatments with rank 1 are the most desirable.

### 2.5. Effect of Fertilization on Grain Yield—N Dose Optimization

According to the ANCOVA results (STY as covariate), the GY of long-strawed wheat varieties was significantly affected by fertilizer treatment (d.f. = 6, F = 6.4, *p* < 0.001). The productivity of short-strawed wheat varieties was also significantly affected by fertilizer treatment (d.f. = 6, F = 6.6, *p* < 0.001) (Table 1).

In the case of long-strawed varieties, the lowest GY was recorded in the control. The application of FYM to the preceding crop, compared to the control, resulted in a slight increase in GY, while no statistically significant difference was observed between FYM and the other fertilized treatments (Table 1). For short-strawed wheat varieties, the unfertilized control also provided the lowest GY, while application of the FYM to the preceding crop resulted in significantly higher GY (+800 kg ha^−1^ in comparison with control). The highest GY was provided by the FYM+NPK treatment, but no statistically significant difference was recorded between NPK2 and FYM+NPK treatments (Table 1). 

**Figure 5:**
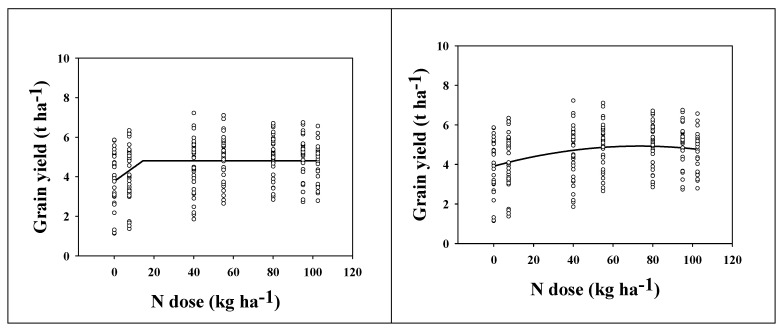
Grain yield (t ha^−1^) of long-strawed varieties as affected by N dose (kg ha^−1^). The data come from 9 seasons (1961–1981). (**Left**) data interleaved with the linear-plateau model. (**Right**) data interleaved with the quadratic model. The shoulder point of the LP model (**left**) occurred at the dose of 15 kg ha^−1^ N (4.8 t ha^−1^). According to the quadratic model (**right**), the maximal yield occurred at the dose of 73 kg ha^−1^ N (4.9 t ha^−1^).

For the purpose of fertilization optimization, two non-linear models were used: the linear-plateau (LP) and quadratic models. For long-strawed varieties (1961–1981, nine seasons), the shoulder point of the LP model occurred at a dose of 15 kg ha^−1^ N, corresponding with a yield of 4.8 t ha^−1^ (Figure 5, left). According to the quadratic model, the maximal yield occurred at a dose of 73 kg ha^−1^ N, corresponding with a yield of 4.9 t ha^−1^ (Figure 5, right, y = −0.0002x^2^ + 0.0278x + 3.9106). As the results of the LP model can be considered too conservative, and the results of the quadratic model as redundantly high (from the point of view of efficiency, economy, and environment protection), a reasonable recommendation for the optimum N rate should be based on the results of both models (the average value) [23]. In our case, the optimum N dose for the long-strawed wheat varieties occurs at 44 kg ha^−1^ N.

The introduction of short-strawed wheat varieties resulted in a significant increase in grain yield (ANCOVA, straw yield as a covariate factor, *p* < 0.001). The average grain yield of long-strawed varieties (1961–1981, 9 seasons) was 4.6 t ha^−1^, while the average grain yield of short-strawed varieties (1983–2020, 14 seasons) was 6.2 t ha^−1^. 

According to the ANCOVA results (Table 1), the NPK2 treatment (55 kg ha^−1^ N) provided comparable grain yield as the FYM+NPK treatment (102.5 kg ha^−1^ N). The application of the FYM to the preceding crop resulted in GY averaging 800 kg ha^−1^ higher than the control, and the difference was significant. The combined application of FYM and NPK resulted in slightly higher yield (+100 kg ha^−1^) than with the NPK4 treatment, but the difference was not significant.

**Figure 6:**
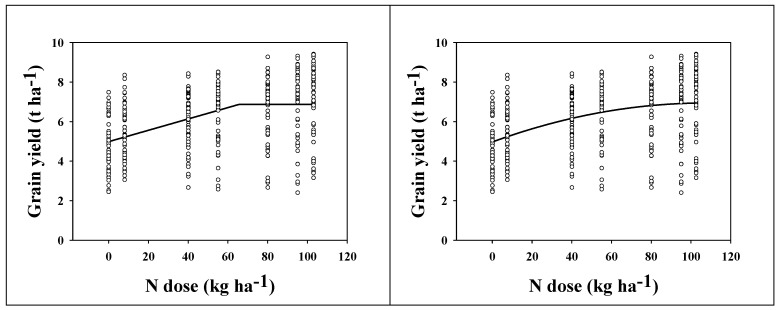
Grain yield (t ha^−1^) of short-strawed wheat varieties as affected by N dose (kg ha^−1^). The data come from 14 seasons (1963–2020). (**Left**) data interleaved with the linear-plateau model. (**Right**) data interleaved with the quadratic model. The shoulder point of the LP model (**left**) occurred at the dose of 66 kg ha^−1^ N (6.9 t ha^−1^). According to the quadratic model (**right**), the maximal yield occurred at the dose of 107 kg ha^−1^ N (7.0 t ha^−1^).

According to the LP model, the shoulder point occurred at the dose of 66 kg ha^−1^ N, corresponding with a GYof 6.9 t ha^−1^ (Figure 6, left). According to the approximation of the quadratic model (Figure 6, right, y = −0.000171x^2^ + 0.0367x + 4.974), the average maximal yield (the local maximum of the quadratic function) was reached at the dose of 107 kg ha^−1^ N, corresponding with a yield of 7.0 t ha^−1^. As mentioned above, this dose of mineral N is redundantly high, and the recommended dose of mineral N for short-strawed varieties grown under comparable soil–climate conditions, as in the LTE, should be approximately 87 kg ha^−1^ (the mean value between 66 and 107 kg ha^−1^ N). This value also provides the most stable GY. 

## 3. Discussion

### 3.1. The Effect of the Weather on GY

Based on the weather analysis, we can say that the conditions at the LTE site are gradually changing. We observed an increase in maximum, average, and minimum temperature as well as precipitation during the year and season for winter crops. In the case of temperatures, this trend is evident throughout the Czech Republic. According to Zahradníček [24], each decade is generally warmer than the previous period, with enhanced warming between 2011 and 2019. In the case of total precipitation, the long-term analysis published by Brázdil [25] proved that the fluctuation of total precipitation is stable in the Czech Republic; negative precipitation trends were recorded between April and June, while positive trends were recorded between July and September. In our paper, we analyzed the relationship between GY and weather parameters of each month of the growing season via the MLR and correlation. The same approaches have been used for analyses of long-term experiments in Müncheberg (Germany) [19] and Rothamsted (UK) [20]. According to the linear regression model used in Germany, the total precipitation during April and July was positively correlated with spring barley GY (when high doses of mineral N were applied), while a negative relationship was found for precipitation in March and temperature in April. In our case, the MLR provided no significant results (n = 32). This comparison is for illustrative purposes only. The weather conditions and weather development throughout time are different at each location and are, therefore, site–specific. The results of one site cannot be generalized to other sites with different soil and climatic conditions. According to the result from Rothamsted [20], the wheat GY is sensitive to mean temperature in November, April, and May, and to precipitation in October, February, and June. Based on the results of this work, the optimum mean November temperature ranges between 6 °C and 7 °C. The April temperature between 8 and 8.5 °C maximizes the wheat GY, while a lower precipitation in June leads to lower yields [20]. In our case, we found a positive relationship between April precipitation and GY, with the optimum ranging between 50 and 60 mm (Figure 4a). Higher or lower precipitation will result in lower than optimum yields. As the mean April precipitation is approximately 32 mm (1954–2020), and the trend of April precipitation is decreasing [25], we can expect that the negative effect of April rainfall on wheat GY will be enhanced in the future. The mean temperature in October was also positively correlated with yields (Figure 4b). Higher October temperatures will beneficially influence seedling emergence and plant germination and provide time for development of stronger plants. Finally, the increasing temperature in February was also evaluated as a factor positively affecting wheat GY (Figure 4c), allowing earlier start of the plant development, provided that there are no other negative aspects, such as higher incidence of pests, diseases, soil acidification, etc.

### 3.2. Yield Stability

The most stable yields of long-strawed wheat varieties (1961–1981) were provided by treatments from the middle range of mineral fertilizers (NPK2 and NPK3, 55–80 kg ha^−1^ N), i.e., neither the lowest nor the highest treatments. The old long-strawed wheat varieties had lower yields than short-strawed varieties [26,27,28] due to better resistance to lodging and diseases, earlier reaching of anthesis, and a longer grain filling period [29]. In the case of short-strawed wheat varieties, the results were the same; the highest yield stability was provided by the same treatments (NPK2 and NPK3). Application of higher doses of mineral N (such as NPK4) generally resulted in slightly higher yields, but according to results of yield stability, also in higher year-to-year variation, i.e., lower stability.

The unfertilized control had the lowest yield stability in both long- and short-strawed varieties. Although the average yields of unfertilized controls increase over time, the wheat in these plots is mainly dependent on the pre-crop effect in terms of nutrients (edge effects were eliminated in the experiment by the harvesting methodology; only the central area of the plot was harvested for experimental purposes). This is confirmed in a study of the long-term experiment at Giessen University (Germany) [22], where similar results were found for an unfertilized control treatment and a similar pattern for PK treatment (without mineral N), showing an unprecedented effect of mineral N on yield stability. Analysis of the long-term experiments in Germany also showed a low ability of unfertilized treatments or treatments without mineral N to provide stable yields [30]. According to this study, the yield stability was also negatively affected by omission of mineral potassium, which can support yield stability, especially under ongoing climate change, as this element controls water management in the plant. 

Application of manure alone (without mineral fertilizers—FYM treatment) to the preceding crop resulted in the second lowest yield stability (in both long- and short-strawed varieties). Similar results were published by Macholdt [31]. We assume that the vast majority of the nutrients from FYM, released by the mineralization, were utilized by the preceding potato crop. The rest of the nutrients, together with the expected beneficial effect of FYM on soil parameters [32,33], slightly (and significantly, in short-strawed varieties) increased the GY (when compared to control), but the fluctuation was still high.

### 3.3. The Effect of Fertilization on GY

As mentioned above, the effect of FYM application to the preceding crop influenced wheat GY. For the long-strawed varieties, there was no significant difference between FYM and control, although FYM application resulted in slightly higher yields. For the short-strawed varieties, the effect was significant (Table 1). Manure not only provides nutrients but also has a beneficial effect on soil properties, positively influencing soil microbial fauna and its activity, nutrient turnover, soil organic carbon, and nitrogen content [33,34], and the effect on wheat GY is visible even three years after its application [35]. This synergy (nutrients + effect on soil) is behind why we observed a significant difference in the short-strawed varieties when comparing FYM versus control. Long-strawed varieties had lower overall yield than short-strawed varieties and were less responsive to N because of their lower nutrient requirements (even high fertilizer rates did not significantly increase yields—quite the opposite, as confirmed by LP and quadratic response models). The introduction of short-strawed wheat varieties into the experiment was associated with higher yields but also with higher nutrient requirements. The quantity of nitrogen to achieve optimum yields in short-strawed varieties was about double that of long-strawed varieties (Figure 5), as the recommended dose of mineral N increased from 44 kg ha^−1^ N to 87 kg ha^−1^ N. These recommended doses were calculated according to Hochmuth [23], based on two non-linear models analyzing the response of GY to N doses. These recommendations are site–specific and should be calculated in relation to the soil and climate conditions. Other researchers have published their own recommendations according to the soil–climate conditions of the analyzed experiments [20,36,37,38,39]. Optimum fertilizer application is important for two reasons. Firstly, it must be economic, so that a positive economic return is achieved for the investment in a fertilizer. Secondly, it must be environmentally friendly, as excessive fertilizer application is not only wasteful but also negatively affects the quality of the environment [40,41].

## 4. Materials and Methods

### 4.1. Site Description

The LTE in Prague can be found on the western edge of the city of Prague, the Czech Republic, central Europe, 50°05′15″ N, 14°17′28″ E. According to the Köppen–Geiger climate classification [42], the site is located in a warm-summer continental climate area (Dfb). The LTE in Prague was established in 1954 with the aim of measuringthe long-term effect of different fertilizer treatments (mineral and organic fertilizers, organic manures), crop rotations, and weather conditions on the yield and quality of arable crops and soil properties. The soil type is Orthic Luvisol, formed by diluvial sediments mixed with loess [43]. The topsoil depth is approximately 0.3 m. The standard climatological long-term mean temperature is 8.7 °C, and the precipitation is 490.4 mm (1954–2020, Crop Research Institute meteorological station). The altitude of the site is 370 m a.s.l.

### 4.2. The Long-Term Experiment (LTE) Description

The LTE in Prague is a large-scale field experiment, consisting of five fields (fields I., II., III., IV., and B). The size of one field is 144 × 96 m (cca 1.4 ha). Each field is divided into 96 individual plots. The size of one plot is 12 × 12 m. In each field, the effects of 24 fertilizer treatments are measured, each treatment replicated four times (24 × 4 = 96 plots) in a completely randomized block design. The crop rotation of the fields II., III., and IV. (analyzed in this paper) consists of nine crops, growing in the order alfalfa, alfalfa, winter wheat, sugar beet, spring barley, potatoes, winter wheat, sugar beet, and spring barley. Out of 24 fertilizer treatments, 7 are analyzed in this paper: (1) control (unfertilized since the LTE establishment), (2) farmyard manure (FYM), (3) NPK1, (4) NPK2, (5) NPK3, (6) NPK4, and (7) FYM+NPK (FYM+NPK4 specifically, but the FYM+NPK abbreviation is used in the paper). The doses of mineral fertilizers are: NPK1—40, 48, 96 kg ha^−1^; NPK2—55, 60, 120 kg ha^−1^; NPK3—80, 48, 96 kg ha^−1^; NPK4—95, 60, 120 kg ha^−1^. The mineral N is applied as limestone ammonium nitrate, P as super phosphate, and K as potassium chloride. Mineral fertilizers are spread evenly by hand. The cattle farmyard manure in the FYM and FYM+NPK treatments is applied to the preceding crop, potatoes, at a dose of 15 t ha^−1^ (approximately 75 kg N). As winter wheat is the first crop following, the estimated amount of N available to and utilizable by wheat represents 10%—7.5 kg ha^−1^. From this point of view, the FYM treatment is calculated as a dose of N of 7.5 kg ha^−1^ and the FYM+NPK treatment as a dose of 102.5 kg ha^−1^ N.

In this paper, we analyze 23 seasons in total, when winter wheat followed by potatoes are grown. These seasons are (the year of harvest) 1961, 1962, 1963, 1970, 1971, 1972, 1974, 1975, 1981, 1983, 1984, 1990, 1992, 1993, 1999, 2001, 2002, 2008, 2010, 2011, 2017, 2019, and 2020. Different wheat varieties have been grown in the experiment since 1961. The long-strawed varieties are Pyšelka (1961–1963), Mironovská (1970–1972), and Jubilar (1974–1981). The short-strawed varieties are Juna (1983–1984), Zdar (1990–1993), Samanta (1999), Alka (2001–2002), Barroko (2008–2010), and Mulan (2011–2020). The sowing of the winter wheat occurs in October; the depth of sowing usually ranges from 3 to 4 cm, and the distance between rows is 0.125 m. Pesticides are used if necessary, and growth regulators are never applied.

### 4.3. Data Analysis

For the calculation of correlations, linear and multiple linear regressions, analysis of covariance (ANCOVA), post hoc analyses (Tukey’s HSD test), and graphical outputs, Statistica 14.0 was used (TIBCO Software, Palo Alto, CA, USA). For the calculation of linear-plateau and quadratic models, SigmaPlot 14.5 was used (Systat Software Inc., Chicago, Illinios, USA). The analysis of stability was performed using the StabilitySoft software [44].

## Figures and Tables

**Table 1:** The effect of fertilizer treatments on GY (t ha^−1^) of long- and short-strawed wheat varieties.

	Long-Strawed Var.	Short-Strawed Var.
	GY (t ha^−1^)	
Control	3.8 ^A^	4.7 ^A^
FYM	4.3 ^AB^	5.5 ^B^
NPK1	4.6 ^B^	6.1 ^BC^
NPK2	4.9 ^B^	6.6 ^CD^
NPK3	4.9 ^B^	6.7 ^CD^
NPK4	4.9 ^B^	6.9 ^D^
FYM+NPK	4.7 ^B^	7.0 ^D^

The capital letters following the average GY present the result of the post hoc analysis (Tukey’s HSD test). Average GY with the same letter are not statistically significantly different from each other.

## Data Availability

Not applicable.
